# The Dino Study: Rationale and protocol for a randomized controlled trial of the Incredible Years Dinosaur Program with daily assessments

**DOI:** 10.1371/journal.pone.0330597

**Published:** 2025-09-08

**Authors:** Rabia R. Chhangur, Eleonore H.M. Smalle, Gerdien van der Zwaag, Egon Dejonckheere

**Affiliations:** 1 Department of Developmental Psychology, Tilburg University, Tilburg, The Netherlands; 2 Tilburg Experience Sampling Center, Tilburg University, Tilburg, The Netherlands; 3 Department of Medical and Clinical Psychology, Tilburg University, Tilburg, The Netherlands; Public Library of Science, UNITED STATES OF AMERICA

## Abstract

**Background:**

Children with conduct problems vary considerably in how they respond to behavioral interventions. Although group-based, child-focused programs are increasingly implemented, research still relies on retrospective parent or teacher reports and group-level outcomes. These traditional approaches often obscure individual differences in treatment response and reduce the potential for individualized behavioral support tailored to each child’s unique profile. The Dino Study—Daily Intervention-based research on Nurturing Opportunities—introduces the Incredible Years Dinosaur Program in the Netherlands for children aged 4–8 with conduct problems. This evidence-based program, grounded in cognitive-behavioral principles, aims to strengthen children’s emotional regulation, social skills, and problem-solving through structured, play-based group sessions. However, little is known about mechanisms of change in children’s daily life, particularly how emotional and behavioral improvements unfold within and across individual children during intervention. This study evaluates the program’s effectiveness in reducing externalizing behavior and promoting cognitive and prosocial functioning, and explores daily variability in children’s emotional and behavioral responses using a daily diary design.

**Methods:**

This protocol outlines a randomized controlled trial with a waitlist control condition. A total of 120 children will be randomly assigned (1:1) to the intervention or waitlist group. Participants will be recruited via professional institutions and primary schools in both preventive and clinical contexts. In addition to pre- and post-assessments, Intensive Longitudinal Data (ILD) will be collected to capture daily fluctuations in mood, behavior, and family interactions before, during, and after the intervention. This design enables analysis of group-level effects as well as intra-individual change patterns.

**Expected outcomes:**

Findings will offer novel insights into how children respond individually to structured interventions in real-life settings and inform more personalized, ecologically valid approaches to early behavioral support.

**Trial registration:**

ClinicalTrials.gov (NCT07051642).

## Introduction

Over the past decades, behavioral interventions for young children have primarily focused on improving parenting practices, based on the assumption that child outcomes are best enhanced by modifying caregiver behavior [1,2]. While such parent-directed approaches have yielded meaningful results, they also highlight a striking limitation: the relative lack of focus on the child as an active agent in the process of change [3,4]. This is particularly problematic for young children with conduct problems, as their heightened difficulties with emotion regulation, social functioning, and adaptive coping make them especially vulnerable in everyday situations [5,6]. At the same time, not all children with behavioral difficulties require the same level of support, highlighting the need for an individualized approach. While some demonstrate resilience and adapt effectively to everyday challenges with limited external support, others appear more sensitive to contextual stressors and benefit from structured, consistent behavioral guidance. However, large-scale intervention programs rarely account for individual differences in responsiveness, and instead evaluate outcomes based on group-level averages [7,8]. Such an approach risks overlooking children who deviate from the average trajectory, including those who derive substantial benefit from the intervention, as well as those who experience little to no improvement. This issue has been recognized in clinical research, prompting a shift toward evaluating personalized intervention outcomes, investigating who benefits most under which conditions, and uncovering the mechanisms by which change occurs [9].

Recent methodological advances offer tools to better capture individual variability in intervention outcomes. In particular, daily diary approaches—drawing on intensive longitudinal data (ILD)—allow researchers to examine short-term fluctuations in children’s emotional and behavioral states across everyday contexts [10,11]. These methods enable a more dynamic and ecologically valid understanding of how interventions are experienced in real time. While variability in child outcomes has been described in earlier studies, most rely on retrospective parent reports or pre-post comparisons, which mask within-person processes and delay the detection of early signs of change [10,12]. This constrains the development of interventions that are attuned to individual developmental trajectories and responsive to early, intra-individual signals of change.

Emerging insights from the Environmental Sensitivity Framework [13]—a synthesis of multiple theoretical models including Differential Susceptibility [14], Vantage Sensitivity [15], Biological Sensitivity to Context [16], and Sensory Processing Sensitivity [17]—suggest that individuals differ in their responsiveness to environmental influences. Children who display greater emotional reactivity or baseline environmental sensitivity may be more responsive to environmental input, including interventions. That is, children who show more day-to-day variability in affect may benefit more from structured, supportive programs in the environment than their less reactive peers. In the context of behavioral problems, emotionally sensitive children may particularly lack the regulatory tools to manage negative affect, making them more vulnerable to emotional escalation during daily challenges [18]. While the Environmental Sensitivity Framework encompasses heightened responsiveness to both negative and positive input, this sensitivity may initially manifest as greater reactivity to stress or frustration. This is particularly the case for children who have not yet developed adaptive coping strategies. Taken together, these insights highlight the need for intervention studies that not only evaluate overall effectiveness, but also examine individual sensitivity and the dynamic processes and mechanisms through which change occurs.

The address this need, the present study evaluates the effectiveness of the Incredible Years (IY) Small Group Dinosaur Child Program, a structured, child-focused intervention aimed at improving children’s emotional regulation, social functioning, and academic-related skills, and at reducing externalizing behavior [19–21] in the Netherlands. The program includes 18 weekly group sessions using playful, dinosaur-themed materials and has demonstrated effectiveness in both clinical and community contexts across countries such as the United States, Canada, and Spain [22–25]. The primary objective is to assess overall program effects on children’s emotional, social, and cognitive development through a randomized controlled trial. In addition to this broad evaluation, the study also incorporates daily diary methodology to examine real-time fluctuations in children’s emotions and behavior, individual sensitivity profiles, and daily parent–child dynamics throughout the intervention period. Through this dual approach, we aim to identify not only variability in outcomes, but also potential mechanisms of change that unfold in daily life. Importantly, this is the first study to combine a randomized controlled trial of the Dinosaur Program with daily diary assessments. This design captures how children respond to the intervention in their everyday environments.

By collecting daily data on emotions, behaviors, and parent–child interactions, the ILD design not only identifies day-to-day variability but also provides insight into emotional sensitivity, behavioral adaptation, and relational patterns over time. Grounded in the Environmental Sensitivity Framework, the study operationalizes emotional sensitivity as moment-to-moment fluctuations in negative affect prior to the intervention, based on empirical indicators derived from daily diary reports. This framing allows us to explore whether children with higher baseline sensitivity respond differently to the intervention. Beyond identifying individual variability, daily diary data offer a window into potential mechanisms of change. Since interventions such as the Dinosaur Child Program aim to influence not only behavior but also emotion regulation and relational dynamics, it is crucial to understand how these processes unfold in the flow of everyday life. By tracking moment-to-moment affective and interpersonal shifts, we can reveal dynamic pathways of change, such as improved co-regulation or reduced emotional escalation. This process-level insight supports a shift from outcome-based evaluation to more personalized, mechanism-informed intervention strategies. Finally, the study examines how family micro-dynamics contribute to intervention outcomes. Because children’s behavior emerges through daily caregiver interactions, ILD enables analysis of these dynamic exchanges over time. Understanding real-life family processes underlying intervention effects is essential for advancing personalized child mental health care and tailoring support to individual needs the field of personalized child mental health care.

### Study aim and research questions

The overarching aim of this study is to evaluate the effectiveness of the Dinosaur Child Program in reducing externalizing behavior and improving emotional and social functioning in children aged 4–8. Furthermore, the study aims to explore individual variability in intervention response and real-time parent–child dynamics using the daily diary methodology.

Specifically, the following research questions and hypotheses are addressed:

1. **Does participation in the Dinosaur Child Program lead to greater improvements in children’s emotional, social, and cognitive functioning, and reductions in externalizing behavior compared to a waitlist control group?**

*H1*: We hypothesize that children in the intervention group will show significantly more improvement in teacher- and parent-reported social, emotional, and cognitive/linguistic competencies, and significantly greater reductions in externalizing behavior than children in the control group.

2. **Does the intervention lead to reduced emotional variability and fewer daily behavior problems, as captured through daily diary methodology?**

*H2*: We hypothesize that children in the intervention group will exhibit fewer and less intense fluctuations in negative affect (controlled for mean level changes) and fewer moments of defiance, tantrums, or other behavior problems across the day, compared to the control group.

3. **Does the intervention alter day-to-day parent–child dynamics by reducing children’s experience of negative affect and promoting shared positive affect in interaction with their parents?**

*H3*: We hypothesize that children in the intervention group will show fewer escalations of negative affect and more intense shared positive moments with their parents than those in the control group. These effects are expected to be most pronounced in children who show higher baseline emotional sensitivity, operationalized as greater moment-to-moment fluctuations in negative affect prior to the intervention.

## Materials and methods

### Study design

**Fig 1:**
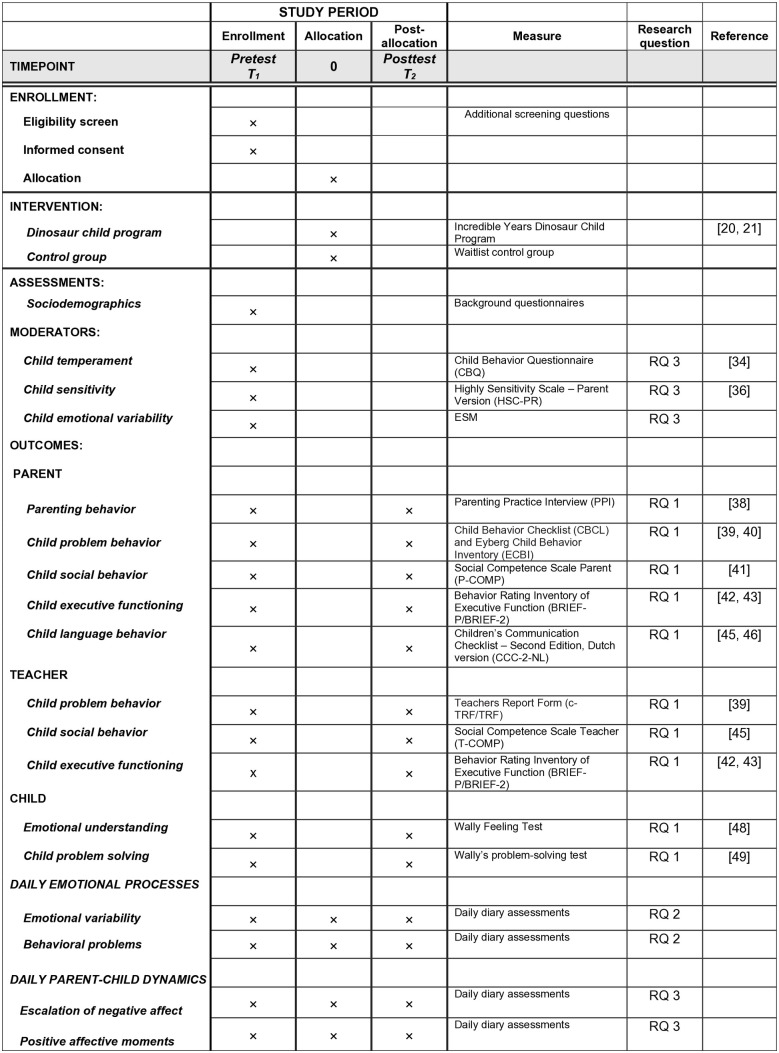
SPIRIT schedule of enrollment, interventions, and assessments in the Dino Study. This figure presents a structured overview of study procedures and assessment timepoints, including enrolment, group allocation, intervention delivery, and outcome assessments. It displays which constructs were measured, by whom (parent, teacher, child), and when (T1 pretest, T2 posttest), and links each measure to the corresponding research question. Daily diary measures are also included to capture within-family processes during the intervention period.

This protocol, which adheres to the SPIRIT 2013 guidelines (see S1 File), describes the Dino Study (Daily Intervention-based research on Nurturing Opportunities), a two-arm randomized controlled trial (RCT) designed to evaluate the effectiveness of the IY Small Group Dinosaur Child Program in Dutch children aged 4–8 years with perceived elevated or (sub)clinical levels of conduct problems [20,21,24]. A total of 120 children and one primary caregiver per family will be enrolled and randomly assigned to either an intervention group (n = 60) or a waitlist control group (n = 60), using a 1:1 ratio. The study assesses both group-level effects and individual variability in treatment response by analyzing intensive longitudinal data (ILD) from daily diaries to capture children’s moment-to-moment negative affect, externalizing behaviors, and daily parent–child interactions. The intervention group will receive the Dinosaur Child Program in small groups across 18 weekly sessions. The control group will receive care-as-usual and will be offered the intervention after posttest assessment. Pretest (T1) and posttest (T2) assessments will be conducted using standardized questionnaires and a brief child interview, with T2 scheduled 19 weeks after T1. In addition, daily diary data on children’s emotional states, behavior problems, and parent–child interactions will be collected via the m-Path application [26] during a one-week baseline period, the 18-week intervention, and a one-week follow-up (i.e., 20 weeks total). Parent and child will complete the diary jointly each evening (Fig 1). Eligibility criteria include: (a) child age between 4 and 8 years at the start of the study (September 2025), (b) parental indication that the child shows elevated or (sub)clinical levels of conduct problems in daily life (e.g., frequent defiance, aggression, or rule-breaking behavior), (c) daily contact between the child and the participating parent, and (c) sufficient fluency in Dutch. Children are excluded if they have an intellectual disability (IQ < 70) or insufficient Dutch language proficiency.

### Recruitment

Participants will be recruited from primary schools as well as preventive and clinical settings in collaboration with two mental health institutions. Information will be shared via email, school newsletters, posters, and individual consultations. Schools previously engaged in the IY training network will be prioritized. Parents who express interest will complete an online registration form and subsequently participate in a video call with trained research staff, who will confirm eligibility and provide detailed information about the study. During this call, digital informed consent will be obtained from parents or legal guardians. When appropriate, children will accompany this call and be given age-appropriate information and asked to provide verbal or digital assent. Participants will have the opportunity to ask questions and may withdraw from the study at any time without consequence.

**Fig 2:**
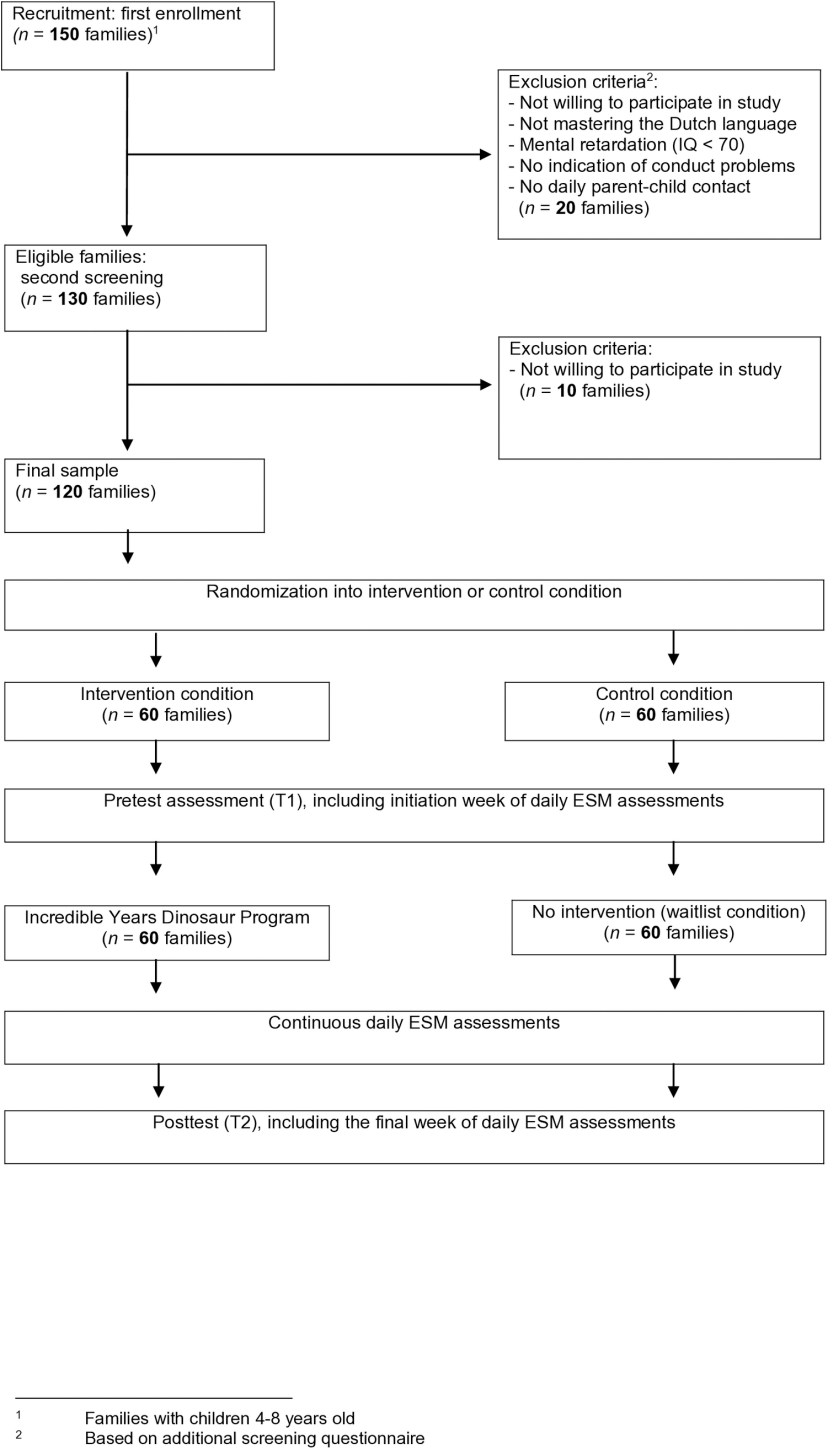
Participant flow and assessment timeline in the Dino Study (CONSORT diagram). The diagram illustrates participant enrollment, exclusion criteria, randomization, and the timing of pretest (T1), the 18-week intervention, and posttest (T2), including daily diary assessments as part of an intensive longitudinal design.

Approximately one month prior to the intervention, families will receive a home visit to establish rapport and conduct baseline procedures. In the intervention group, this visit will be conducted by the child’s future group leader; in the control group, by a trained research assistant. During the visit, children will provide oral assent and complete a brief, age-appropriate interview. Parents will complete baseline questionnaires and receive instructions for installing the m-Path app [26]. An overview of participant flow, including enrollment, randomization, and assessment timing, is shown in Fig 2.

### Power analysis

At the between-person level, an a priori G*Power analysis [27] indicated that 64 participants per group are required to detect a medium effect size (Cohen’s *d* = 0.5), and 26 per group for a large effect size (*d* = 0.8), assuming α = 0.05 and statistical power of 0.80. Previous studies of the IY Dinosaur Child Program have reported effect sizes between 0.70 and 0.81 for improvements in social competence [19], supporting the planned sample size of 120 to allow for potential attrition.

To evaluate intervention effects on intra-individual and dyadic dynamics via daily diaries, simulation-based power calculations were performed. These simulations examined time by condition (intervention vs. control) interactions in multilevel models, with rolling-window correlations nested within participants/families [28]. With 140 time points per child/ family (i.e., 7 days * 20 weeks), statistical power is expected to exceed 80% for detecting small interaction effects (β = 0.005).

### Randomization

This single-blinded RCT uses a randomized allocation procedure with a 1:1 allocation ratio to assign participants to either the intervention or waitlist control group. An independent researcher will generate the random allocation sequence using a computer-based random number generator. The personnel responsible for enrolling participants and assigning them to interventions will not have access to the random allocation sequence until after the baseline assessment is completed, at which point parents will be notified of the group assignment.

### Data collection and procedures

A detailed overview of measures by informant and timepoint is provided in Fig 1 (SPIRIT schedule). All standardized pretest and posttest measures have demonstrated good psychometric properties. The diary items were adapted from validated instruments (e.g., PANAS-C, externalizing spectrum scales, parent-child relationships [29–32]) and tailored for daily use in young children. While validation of daily diary in early childhood is still emerging, this study contributes to ongoing efforts to evaluate ILD methods in child intervention research.

Data collection spans three phases: pre-intervention (T1), intervention, and post-intervention (T2). During T1 (September 2025), parents and teachers complete online questionnaires. The home visit includes child assent, a brief emotion regulation task, and setup of the m-Path app. From the baseline period through the 18-week intervention and one-week follow-up, daily digital assessments will be completed jointly by parent and child each evening, focusing on emotions, behaviors, and family interactions. To encourage consistent participation, families can earn up to €50 per family as incentives, distributed across key milestones: €10 for completing the pretest questionnaires, approximately €25 for daily questionnaire completion during the study period (with €0.15 per completed daily questionnaire), and €15 for completing the posttest questionnaires. Participation progress is tracked via the m-Path app, and payments will be made after study completion based on participation levels.

The study procedure has been approved by the Ethics Review Board of Tilburg University. All participating parents will provided written informed consent for their own and their child’s participation, in accordance with the Declaration of Helsinki. Supporting materials, including the research proposal submitted for ethical review, the information letters for parents and teachers, and the informed consent form, are available in the supporting information (see S2 File). To protect the privacy of participating children and their parents, all shared data will be de-identified and stored in a secure repository (TiU Dataverse). Data will not be openly accessible but can be obtained upon reasonable request. Researchers who wish to access the data must submit a research proposal that falls outside the scope of the original research line, acknowledge the data source, and sign a data-sharing agreement. The company associated with one of the authors (m-Path Software) had no role in the study design, data collection and analysis, decision to publish, or preparation of the manuscript.

#### Timeline.

Participant recruitment will start in August 2025 and is expected to be completed by October 3, 2025. Intake interviews—including eligibility screening, explanation of the study procedures, and digital informed consent—will take place between September 1 and October 3, 2025, via scheduled video calls. Baseline assessments (T1), including home visits, parent questionnaires, and child interviews, will occur between September 15 and October 17, 2025. The intervention is scheduled to begin in the week of October 20, 2025, and will continue for 18 sessions through the week of March 9, 2026. Posttest assessments (T2), including follow-up home visits and questionnaires, will be conducted between March 16 and April 17, 2026. This timeline ensures a 5-week pretest period, an 18-week intervention period, and a 5-week posttest window. Study results are expected to be available by September 2026 and will be submitted for publication, presented at conferences, and reported in ClinicalTrials.gov.

### Intervention

#### Incredible Years Small Group Dinosaur Child Program.

The intervention group will receive the IY Small Group Dinosaur Child Program, a structured, manualized cognitive-behavioral intervention originally developed to support young children (ages 3–8) with conduct problems [20,21,24]. The program is designed to enhance emotional regulation, prosocial behavior, and problem-solving abilities through developmentally appropriate, interactive group-based sessions. Although initially developed for children with externalizing difficulties, it is now widely applied in broader populations, including children with internalizing difficulties such as social-emotional or attentional challenges [33]. In this study, the intervention will target children aged 4–8 years who exhibit elevated levels or (sub)clinical levels of conduct problems.

The program will be delivered in ten small groups, each consisting of 5–6 children. Each group will be facilitated by one certified group leader, using the Dutch translation and adaptation of the original IY protocol. Sessions will be held weekly for 18 weeks, with each session lasting approximately two hours. The curriculum will include storytelling with puppets, video modeling, role-play exercises, group discussions, and cooperative games. These elements are intended to foster emotional awareness, frustration tolerance, interpersonal problem-solving, and socially appropriate behavior. Parents in the intervention group will not receive a parallel parent training during the study period.

All groups will be led by one of six certified IY group leaders. Four of these professionals will be licensed child therapists affiliated with Psychologenpraktijk Timmers or Stichting Kumbaya, and two will be affiliated with Tilburg University. Prior to the start of the trial, all trainers will have completed the official IY certification process, which includes delivery of a full intervention cycle and a successful video-reviewed evaluation of fidelity and delivery quality.

#### Control condition.

Participants assigned to the control condition will be placed on a waitlist and will not participate in any structured group-based intervention during the study period. They will retain access to standard care and community-based services as needed, but will not receive any elements of the IY program during the data collection phase. Information on additional support received will be systematically collected via parental questionnaires.

After completion of the posttest assessment (T2), families in the control group will be offered the opportunity—on a voluntary basis—to participate in the IY Dinosaur Program. This post-trial intervention will be delivered by the same certified group leaders and institutions involved in the study (Tilburg University, Psychologenpraktijk Timmers, or Stichting Kumbaya), but will not be part of the research protocol. This design allows for a controlled comparison of intervention and non-intervention conditions, while ensuring equitable access to support after study completion.

#### Fidelity monitoring.

To ensure high-fidelity implementation, group leaders will follow a standardized session protocol and complete adherence checklists after each session. Fidelity will be further supported through regular supervision meetings with certified IY consultants. Prior to the start of the trial, all group leaders will have completed the formal IY certification process, including a video-reviewed delivery of the full program. Any deviations from the protocol will be documented and reported in the final study manuscript.

### Moderators

To explore individual variability in response to the intervention, several child-level moderators will be assessed prior to the start of the intervention (T1). These include emotional sensitivity, temperament traits, general sensitivity, and executive functioning. In addition, fluctuations in daily emotional functioning will be captured through ILD. These moderators will be examined as potential predictors of differential treatment effects.

#### Child temperament.

Child temperament will be assessed using the Child Behavior Questionnaire – Very Short Form (CBQ-VSF), a widely used parent-report instrument [34]. The CBQ-VSF contains 36 items rated on a 7-point Likert scale ranging from 1 (“extremely untrue”) to 7 (“extremely true”). Items cover multiple dimensions of temperament; in the current study, the Negative Affectivity subscale (e.g., “My child gets quite frustrated when prevented from doing something s/he wants to do”) will be used as a moderator. A composite score will be calculated as the mean of the subscale items. The CBQ-SF has demonstrated good internal consistency and construct validity in prior studies [35].

#### General child sensitivity.

The Highly Sensitive Child – Parent Report (HSC-PR) is a 12-item parent questionnaire that assesses environmental sensitivity in children [36]. Items are rated on a 7-point scale (1 = “not at all” to 7 = “very much”). A sample item is: “My child notices subtle smells or sounds that others do not.” Total scores will be calculated by averaging all items, with higher scores indicating greater sensitivity. The HSC-PR has shown good reliability and factor structure in recent studies [37].

#### Child emotional variability.

Emotional variability will be operationalized using parent–child dyadic daily assessments collected via the m-Path app [26]. Each evening, parents and children will complete a short diary survey including 2–3 emotion-related items (e.g., “Did your child experience strong emotions today?”) on a 5-point scale (1 = “not at all” to 5 = “very much”). Variability will be calculated based on within-person standard deviations, and changes over time will be explored using a moving window approach [28].

### Outcome measures

This study includes both group-level outcomes, assessed through pre- and post-intervention surveys, and micro-level measures, assessed through daily diaries, to evaluate the effectiveness of the Dinosaur Child Program across emotional, behavioral, and cognitive domains. Outcomes are collected via questionnaires from three informants (parents, teachers, and children), and supplemented by daily parent–child dyadic daily diary reports. Measures are aligned with the three main research questions. Primary outcomes are used to assess intervention effects at the group level (RQ1), while secondary outcomes capture dynamic emotional and behavioral processes within children (RQ2) and their families (RQ3).

#### Parent-reported outcomes.

***Parenting behavior (Explanatory outcome; RQ1).*** Parenting practices will be measured using the Parenting Practices Interview (PPI) [38]. The PPI includes 73 items rated on a 6-point Likert scale (1 = “never” to 6 = “always”) and covers domains such as positive involvement, inconsistent discipline, and harshness. A sample item is: “When your child misbehaves, how often do you give up trying to discipline him/her?”. Subscale scores will be used to explore potential changes in parenting behavior. Although parents are not enrolled in a formal parenting program, those whose children receive the Dinosaur intervention are indirectly exposed to parenting principles from the IY program. Through weekly letters, reading assignments, and homework exercises derived from the parent book used in the IY parent training, caregivers are encouraged to apply positive parenting strategies at home. The PPI was specifically developed to assess parenting behaviors targeted by the IY parent intervention

***Child externalizing behavior (Primary outcome; RQ1).*** The Child Behavior Checklist (CBCL) will be used to assess children’s behavioral and emotional problems from the parent’s perspective [39]. The CBCL includes 113 items rated on a 3-point scale (0 = “not true”, 1 = “somewhat true”, 2 = “very true”). The Externalizing Problems composite will serve as the primary indicator of behavioral change. To complement this, the Eyberg Child Behavior Inventory (ECBI) will be administered [40]. The ECBI consists of 36 items (e.g., “Refuses to go to bed”) rated for both frequency and problem severity.

***Child social behavior (Primary outcome; RQ1).*** Social competence will be measured using the Social Competence Scale – Parent version (P-COMP) [41]. This 12-item scale uses a 5-point Likert format (1 = “not at all” to 5 = “very well”) to evaluate skills like cooperation, self-control, and empathy. A sample item is: “How well does your child get along with other children?”.

***Executive functioning (Primary outcome; RQ1)***. Parents will complete the Behavior Rating Inventory of Executive Function, Second Edition– Parent form (BRIEF-2) [42,43]. This version includes 60 items rated on a 3-point Likert scale (1 = “never”, 2 = “sometimes”, 3 = “often”) and assesses executive functioning in daily life at home. A sample item is: “Has trouble remembering things, even for a few minutes.” The Global Executive Composite (GEC) score will be used at the primary outcome. The BRIEF-2 has demonstrated strong psychometric properties in both clinical and normative samples [44].

***Child language behavior (Primary outcome; RQ1).*** The Children’s Communication Checklist – Second Edition, Dutch version (CCC-2-NL) [45,46] will be used to assess the child’s communicative/language functioning from the perspective of the parent or caregiver. The CCC-2-NL consists of 70 items covering structural language skills (such as syntax and semantics) as well as pragmatic aspects of communication (e.g., inappropriate initiation, use of context, nonverbal communication). Items are rated on a 4-point scale (0 = “less than once a week or never” to 3 = “several times a day or always”), providing standardized composite scores to identify potential communication difficulties. The General Communication Composite (GCC) derived from the CCC-2-NL will serve as the primary indicator of change in child communicative behavior.

#### Teacher-reported outcomes.

***Child externalizing behavior (Primary outcome; RQ1).*** The Teacher’s Report Form (TRF) will be used to assess behavioral functioning in the school setting [39]. The TRF mirrors the CBCL and includes 120 items with the same 3-point scale. The Externalizing composite score will be used as a primary outcome.

***Child social behavior (Primary outcome; RQ1).*** Teachers will also complete the Social Competence Scale – Teacher version (T-COMP) [47]. This version includes items similar to those in the parent version and evaluates the child’s social functioning at school. T-COMP scores will be analyzed alongside parent reports to assess cross-contextual consistency in social competence.

***Child executive functioning (Primary outcome; RQ1).*** Teachers will complete the Behavior Rating Inventory of Executive Function, Second Edition – Teacher Form (BRIEF-2) [42,43]. This version contains 61 items, also rated on a 3-point Likert scale, and evaluates the child’s executive functioning in the school setting, including classroom behavior, task focus, and emotional regulation. The Global Executive Composite (GEC) score will be used to parallel the parent-reported outcome and enable comparison across contexts.

#### Child-assessed outcomes.

***Emotional understanding (Primary outcome; RQ1).*** Children’s emotional awareness will be assessed using the Wally Feeling Test [48], a structured task in which children identify and explain emotions depicted in cartoon vignettes.

***Problem-solving skills (Primary outcome; RQ1).*** Children will complete the Wally Problem Solving Test [49], a structured measure in which social dilemmas are presented in an illustrated format. Children are asked to generate possible solutions for each scenario. Responses are coded for quality and diversity, providing insight into cognitive-behavioral competencies such as perspective-taking, emotional regulation, and flexible problem solving—key targets of the intervention.

#### Daily diary-based outcomes.

***Emotional variability and behavior problems (Secondary outcomes; RQ2).*** To capture within-person variability in emotional and behavioral functioning, daily diary surveys will assess both emotional functioning and behavior problems, completed each evening by the parent–child dyad using the m-Path app [26]. Emotional variability will be measured using a set of seven momentary emotion items derived from the Positive and Negative Affect Schedule for Children (PANAS-C) [29] and the original PANAS [30]. Children will indicate how they felt “at the moment” on a 7-point scale ranging from 1 (*not at all*) to 7 (*very much*), for the emotions proud, happy, calm, ashamed, sad, angry, and tense. To enhance developmental appropriateness and alignment with the Dinosaur Child Program, each response option is accompanied by a visual cue: a small-to-large image representing the intensity of each specific emotion, consistent with the emotion cue cards used during the intervention. Behavior problems will be assessed with three items reflecting hostility-related externalizing behaviors, adapted from the externalizing spectrum framework [31]. These include losing one’s patience, saying something mean to someone, and being angry or aggressive towards someone. Responses will be recorded on a 5-point Likert scale ranging from 1 (*not at all*) to 5 (*very often*), allowing for sensitive detection of daily fluctuations in child behavior over time.

***Parent–child affective dynamics (Secondary outcomes; RQ3).*** To examine daily relational dynamics between parents and children, both members of the dyad independently complete a daily diary assessing shared emotional moments, negative interactions, emotion regulation, and time spent together. These items were adapted from validated measures of momentary relationship quality [32] and reflect shared emotional experiences, conflict-related emotions, and individual efforts at emotion regulation. This dyadic structure allows for the analysis of affective synchrony, divergence, and co-regulatory processes, which are central to understanding how the intervention impacts family functioning over time. Parents report whether they experienced nice moments with their child (“I had some nice moments with my child today.”) and whether they felt angry toward their child (“I got angry at my child today.”). These items are rated on 5-point Likert scales using either an intensity format (1 = *not at all* to 5 = *very much*) or a frequency format (1 = *not at all* to 5 = *very often*). In addition, a contextual control item is included to assess the amount of parent–child contact: “How many hours were you with your child today (excluding sleep time)?” (ranging from 0 to 24 hours). Children complete a mirrored set of items: they report whether they had nice moments with their parent, whether they got angry at their parent, how difficult it was to behave well, and what they did to manage their emotions if they felt angry. The regulation item includes a multiple-choice question offering strategies that are taught during the Dinosaur Child Program (e.g., taking deep breaths, doing the “turtle pose,” or thinking of something fun), along with an open-ended “other” option. Ratings use the same Likert formats as in the parent version to ensure comparability.

By collecting matched daily reports across 20 weeks of measurement, this dyadic ILD approach enables rich time-series analyses of both shared and diverging perceptions within the family. It allows the study to examine not only whether parent–child dynamics improve overall, but also whether these improvements are co-experienced and co-regulated — for example, whether decreases in child-reported anger correspond with reductions in parent-reported conflict, or whether increases in positive moments are felt mutually or asymmetrically. The inclusion of contextual time data enables the control of fluctuations in contact intensity, helping to interpret changes in affective dynamics relative to daily routines and caregiving structures.

### Statistical analysis

All data will be analyzed using R [50] and Mplus [51]. Each family will be assigned a unique study ID to ensure anonymized data handling. Statistical analyses will follow a staged approach, aligned with the study’s three hypotheses. Missing data will be handled using full information maximum likelihood estimation within Latent Change Score (LCS) and multilevel models, minimizing bias due to attrition or intermittent missingness. Sensitivity analyses, including intention-to-treat analyses that incorporate all randomized participants regardless of adherence or dropout, will be performed to assess the robustness of the findings. Additional subgroup analyses exploring moderators such as child age, gender, and baseline severity will be conducted to evaluate consistency of intervention effects.

#### Preliminary analyses.

To evaluate baseline equivalence between the intervention and control groups, independent samples t-tests (or Mann–Whitney U tests for non-normally distributed variables) will be conducted on demographic characteristics, baseline parenting behavior, and child behavioral outcomes. Any baseline differences that reach statistical significance will be included as covariates in subsequent models.

#### Hypothesis 1: Group-level behavioral effects.

To assess whether participation in the Dinosaur Child Program leads to greater improvements in children’s emotional, social, behavioral and cognitive functioning, LCS models will be estimated in Mplus for each primary outcome domain: child externalizing behavior, social competence, emotional understanding, and cognitive functioning. Outcomes will be analyzed separately for parent-reported, teacher-reported, and child-assessed measures. LCS models estimate change over time using latent difference scores, thereby distinguishing true change from measurement error. Intervention condition will be tested as a predictor of pre-to-post change examine differential improvements between the intervention and control group. Each latent change score model will estimate the average change over time, interindividual variability in change, and the effect of group assignment on these change scores. Bonferroni-adjusted significance thresholds will be applied to control for multiple comparisons across outcome domains.

#### Hypothesis 2: Daily variability and behavior problems (ILD-based).

To examine whether the intervention reduces children’s daily emotional variability and behavior problems, multilevel time-series analyses will be conducted on daily parent–child diary data. Daily reports will include ratings of negative affect (e.g., irritability, sadness) and behavior problems (e.g., defiance, tantrums). Repeated observations will be modeled as nested within individuals to capture both group-level trends and within-person variability over time. Fixed effects for time, group, and their interaction will be included to test for differential change trajectories across conditions. In addition, moving window techniques will be employed to capture changes in intra-individual fluctuations and identify evolving temporal patterns of variability throughout the intervention period.

#### Hypothesis 3: Parent–child dynamics (dyadic ILD-based).

To evaluate whether the intervention enhances the emotional quality of daily parent–child interactions, multilevel time-series analyses will be applied to dyadic daily diary data. These models will examine daily reports of shared positive affect and emotional escalation during conflict, with repeated measures nested within families. Time-by-condition interaction terms will be used to assess whether trajectories of affective dynamics differ between the intervention and control groups. Moving window techniques will be used to derive fine-grained temporal indicators of affective synchrony and escalation, allowing for a dynamic examination of how relational processes evolve across the intervention period. This analytic approach offers a detailed understanding of whether the intervention promotes more stable and positive emotional exchanges between parents and children.

## Discussion

Although behavioral interventions such as the IY Dinosaur Program have demonstrated positive effects in supporting young children with conduct problems, substantial heterogeneity in outcomes remains [19,21,24,33]. This study protocol addresses the critical question of what works, for whom, and what are mechanisms of change? By integrating an RCT with a high-frequent ILD design, the Dino Study introduces a novel approach that allows for the examination of both average treatment effects and individual variability in real-life settings. A key innovation of this protocol lies in its combination of group-level standardized assessments and fine-grained, real-time data collection, as well as its comprehensive assessment of outcomes across emotional, behavioral, and cognitive domains. Most intervention studies rely on retrospective reports and pre–post designs, which obscure intra-individual dynamics and parent–child processes that unfold over time [7,8].

ILD collection enables the daily tracking of behavioral and emotional functioning, offering a dynamic, ecologically valid understanding of how children and families respond to structured interventions. Diary and EMA methods reduce retrospective bias and allow for the detection of within-person change processes—such as immediate responses to intervention—that traditional assessments may overlook [52]. This approach provides insight not only into whether a program works, but also into how change unfolds, for whom it is most effective, and under which contextual or emotional conditions. The study is grounded in the Environmental Sensitivity Framework [13], which posits that children differ in their responsiveness to environmental input. By examining moderators such as temperament, executive functioning, and affective variability, the study explores the potential for differential responsiveness to supportive interventions. For example, children who show higher daily emotional reactivity at baseline may be particularly receptive to structured, consistent feedback.

Another important contribution of this study is its focus on affective parent–child dynamics as both an outcome and potential mechanism of change. Rather than limiting evaluation to child behavior alone, daily surveying will provide insights into shared emotional experiences and daily escalation or co-regulation patterns. This level of resolution allows researchers to move beyond the “omnibus effect” typically reported in large-scale RCTs and begin unpacking the micro-level processes that may underlie successful behavior change. At the same time, the study acknowledges the inherent complexity of such data: the intervention is a multi-component program, and causal specificity regarding which elements are most effective may remain difficult to determine.

The waitlist control design allows for strong internal comparisons, but it does not isolate non-specific intervention effects such as increased attention or structure. Furthermore, although the ILD approach is developmentally adapted and only administered once daily, the potential burden on families may still affect compliance and data quality. These limitations are offset, however, by the study’s strengths: the use of certified trainers, implementation in both clinical and preventive settings, and integration of multi-informant data sources, including parent, teacher, and child assessments.

Ultimately, the Dino Study contributes to the emerging movement toward precision prevention in child mental health care. By capturing day-to-day changes and individual differences in responsiveness, this study may help identify subgroups of children for whom structured interventions are especially effective. In doing so, it moves the field closer to personalized, data-informed approaches that respect the complexity of child development and the diversity of family contexts.

### Trial registration

The protocol of this study is registered at ClinicalTrials.gov with identification number NCT07051642 on July 9^th^, 2025. Protocol version 1, date 09.07.2025. The first participant has not yet been recruited. Additional details about the trial registration are presented Table 1.

**Table 1:** Overview of measurements.

Data category	Information
Primary registry and trial identifying number	ClinicalTrials.gov
	NCT07051642
Date of registration in primary registry	July, 9th, 2025
Secondary identifying numbers	TSB_RP2172
Contact for public queries	Rabia R. Chhangur e-mail: r.r.chhangur@tilburguniverisity.edu
Contact for scientific queries	Rabia R. Chhangur e-mail: r.r.chhangur@tilburguniverisity.edu
	Tilburg University, Tilburg, The Netherlands
Scientific title	The Dino Study: Rationale and study protocol for a randomized controlled trial evaluating the Incredible Years Dinosaur Program with daily assessments
Countries of recruitment	The Netherlands
Health condition(s) or problem (s) studied	Behavior problems in children, parent-child relations, oppositional behavior, executive functioning, and social and language skills
Intervention(s)	Active comparator: Incredible Years Dinosaur Program
	Control group: Waitlist control condition
Key inclusion and exclusion criteria	Ages eligible for study: child, adult, older adult
	Sexes eligible for study: both
	Accepts healthy volunteers: yes
	Inclusion criteria: Dutch-speaking children aged 4–8 years, at least one Dutch-speaking parent willing and able to participate in the study
	Exclusion criteria: intellectual disability in the child (IQ < 70), child lives primarily in a different household during weekdays, parent or child does not have sufficient Dutch language proficiency
Study type	Interventional
	Allocation: randomized, 1:1 assignment
	Masking: single (outcome assessor)
	Primary purpose: treatment
	Phase: not applicable
Estimated study start date	September 2025
Estimated primary completion date	April 2026
Target sample size	120
Recruitment status	Not yet recruiting
Primary outcome(s)	Change in social competence (parent and teacher reports) Change in externalizing behavior (parent and teacher reports) Change in executive functioning (parent and teacher reports) Change in language and communication skills (parent reports) Change in child-assessed emotion understanding and problem solving
Secondary outcome(s)	Daily dynamic emotional processes (ILD-based)
	Daily behavioral processes within families (Dyadic ILD-based)

## Supporting information

S1 File SPIRIT checklist. (DOCX)

S2 File Research proposal for ethics review, information letters for parents and teachers, and informed consent form. (PDF)
