# Correction: The Dino Study: Rationale and Protocol for a Randomized Controlled Trial of the Incredible Years Dinosaur Program with Daily Assessments

**DOI:** 10.1371/journal.pone.0339314

**Published:** 2025-12-23

**Authors:** The PLOS One Staff

## Notice of Republication

This article was republished on December 5, 2025, to correct the author order. The author order needs to change from

Rabia R. Chhangur, Eleonore H.M. Smalle1, Egon Dejonckheere, and Gerdien van der Zwaag

to

Rabia R. Chhangur, Eleonore H.M. Smalle, Gerdien van der Zwaag, and Egon Dejonckheere

## Supporting information

S1 File Originally published, uncorrected article. (PDF)

S2 File Republished, corrected article. (PDF)
