# Supplementary material for: The Dino Study: Rationale and protocol for a randomized controlled trial of the Incredible Years Dinosaur Program with daily assessments
Source: PLoS One. 2025 Sep 8;20(9):e0330597. doi: 10.1371/journal.pone.0330597 (PMC12416688; doi:10.1371/journal.pone.0330597)
Supplement: S2 File — (PDF) [file pone.0330597.s002.pdf]

The application form: **G.E.D. Started!** (GDPR, Ethics, and Data Management): TBS Ethics Review Board – Case: TBS_RP2172

Personal Information

School:
Which school do you want to submit your application to?
TBS

Department
Which Department do you work for?
Development Psychology

Information principal investigator
Please select your title(s)
☐ Prof. ☐ Ir.
☑ Dr. ☐ Mr.

Project duration
Please indicate the proposed start date and proposed end data of the project.
13-06-2025 - 12-06-2026

Funding organization
Is there a funding organization?
☐ There are no funding organizations
☑ The funding organizations are: NWO

Student research
Is this research project conducted by students?
☑ No

Language
Are all documents in English?
☑ No

Which TiU researcher holding a PhD is responsible for this application?
Rabia Chhangur

General Information

Title
Dino Study: **D**aily **I**ntervention-based research for **N**urturing **O**pportunities

Background
Intervention studies often prioritize parents over directly supporting young children with behavioral challenges. However, children respond differently to parent-focused interventions—some adapt easily, while others need targeted support. Despite this, large-

scale child-focused interventions rarely examine real-time individual differences using Experience Sampling Methods (ESM).

Traditional research often relies on group averages, which can obscure significant variability and limit personalized interventions. Measuring behavioral change in young children is particularly difficult due to their developing self-regulation. To address this, this study introduces the Incredible Years Dinosaur Child Program in the Netherlands. Through a randomized controlled trial (RCT), it will assess the program's effectiveness while integrating ESM to track individual responses. These findings could inform future child mental health interventions.

The Incredible Years (IY) Dinosaur Child Program is an evidence-based intervention aimed at improving self-regulation and social-emotional skills in children aged 4–8 with conduct problems. The program, implemented globally, consists of 18 weekly sessions using playful, dinosaur-themed activities to engage children.

Traditional child-focused research often relies on retrospective parent reports or structured assessments, which lack ecological validity and overlook real-time behaviors. ESM addresses these gaps by capturing daily emotional and behavioral fluctuations, offering a nuanced view of intervention effectiveness.

Parent-child interactions shape children's behavior, making it essential to study real-time family dynamics. By analyzing moment-to-moment variability, this study will provide insights into optimizing interventions for different children and families, advancing personalized approaches in child mental health.

Research question(s)

Program's effectiveness on group level

1. Do children in the intervention group improve their social, emotional, and academic competencies more effectively than those in the control group?
2. Do children in the intervention group reduce problem behavior more effectively than those in the control group?

Program's effectiveness on individual level

1. Does the intervention reduce within-day fluctuations in negative affect, leading to greater emotional stability (fewer mood swings) compared to the control group?
2. Does the intervention decrease the frequency and intensity of challenging moments (e.g., tantrums or defiance) between the child and their parent compared to the control group?

Program's effectiveness on family micro-dynamics

1. Does the intervention reduce the development of negative affect in children at micro-timescales, leading to fewer challenging moments and more shared positive moments between the child and their parent compared to the control group?

2. Does the intervention enhance the development of positive affect in children at micro-timescales, amplifying shared positive moments and reducing challenging moments between the child and their parent compared to the control group?

Study design and methodology

We will conduct a randomized controlled trial (RCT) comparing children receiving the IY Dinosaur Child Program with those in the standard care (waitlist) condition. A total of 120 children (aged 6–8) with (sub)clinical problem behaviors will be recruited. Exclusion criteria include a diagnosed psychiatric or neurological disorder (as reported by the parent), intellectual disability (IQ < 70), insufficient proficiency in the Dutch language, or not living primarily with the participating parent. This sample size ensures sufficient statistical power to detect meaningful effects, particularly with frequent ESM measurements, while also accounting for potential attrition to maintain robust outcomes.

After enrollment, children will be randomly assigned in a 1:1 ratio to either the control group (n = 60) or the intervention group (n = 60). Parents, teachers, and children will complete pretest surveys to establish baseline child behavior, after which the IY Dinosaur Child Program will be implemented. Children in the control group will not receive the intervention but will have access to mental health care and parenting support through regular services if needed. A posttest assessment will take place five months after the baseline assessment.

Throughout the 18-week intervention and for one additional week afterward, parents and children will complete a short daily ESM questionnaire, focusing on shared emotions and behaviors. The primary outcomes will assess the program's effectiveness both at the group level through online surveys and at the individual level through ESM. Secondary outcomes will examine how family micro-dynamics contribute to individual differences in response to the intervention.

Procedures and materials

Participants will receive detailed study information and provide informed consent before randomization into either the intervention or control group. Recruitment and registration will take place between May and July 2025. The pretest will be conducted in August 2025, during which parents and teachers will complete online questionnaires, and children will be interviewed at home. Home visits will be conducted by therapists and researchers from Tilburg University, which will also oversee visits for the control group.

The intervention will begin in the week of September 22, 2025, lasting 18 weeks until February 9, 2026, excluding holidays. Throughout the intervention and in the week before and after, parents and children will complete a short ESM questionnaire between dinnertime and bedtime.

The posttest will take place between February and March 2026, during which parents and teachers will again complete online questionnaires, and children will be re-interviewed at home.

Measurements

Parent-Reported Child Behavior

- Pretest and posttest: Child Behavior Checklist (CBCL; Achenbach & Rescorla, 2001), Social Competence Scale for Parents (P-COMP; Conduct Problems Prevention Research Group, 1995).
- Pretest only: Child Behavior Questionnaire (CBQ; Rothbart et al., 2001), Highly Sensitive Child Scale (HSC-PR; Pluess et al., 2018), Behavior Rating Inventory of Executive Function (BRIEF-2; Gioia et al., 2015).

Teacher-Reported Child Behavior

- Pretest and posttest: Social Competence Scale for Teachers (T-COMP; Conduct Problems Prevention Research Group, 1995), Child Behavior Checklist for Teachers (CBCL-TRF; Achenbach & Rescorla, 2001).
- Pretest only: Behavior Rating Inventory of Executive Function (BRIEF-2; Gioia et al., 2015).

Parent-Reported Parenting Behavior

- Pretest and posttest: Parent Practices Interview (PPI; Webster-Stratton, 1998).

Child Assessments

- Pretest and posttest: Wally Feeling Test (emotion recognition) and Wally Problem-Solving Test (social problem-solving skills) (Denham & Couchoud, 1990).

Ecological Momentary Assessment (ESM)
Parents and children will complete brief daily questionnaires about emotions and behaviors. This method provides real-time insights into parent-child interactions and emotional stability throughout the intervention.

Which tools will be used for data collection and/or data analyzing?
☐ Mturk
☑ Qualtrics
☑ SPSS
☐ Atlas.ti
☑ R
☐ Other
☑ Mplus
☐ None

Scientific and societal relevance

This study addresses a critical gap in intervention research by focusing on real-time individual differences in children's responses to behavioral interventions. Traditional approaches primarily target parents, assuming a uniform impact on children, yet not all children benefit equally. By integrating the Incredible Years Dinosaur Child Program with Experience Sampling Method (ESM) in a randomized controlled trial (RCT), this study provides a nuanced understanding of how young children regulate emotions and behaviors in daily life. Unlike retrospective parent reports or structured lab assessments, ESM captures real-time fluctuations in children's affect and parent-child interactions, improving ecological validity. This approach enables a more personalized understanding of intervention effectiveness, distinguishing between children who naturally adapt and those requiring more targeted support. The findings will contribute to both scientific knowledge and clinical practice by informing more tailored interventions for children with conduct problems, ultimately enhancing their emotional well-being and family dynamics.

Have ethical issues already been assessed?
☑ No

Are you only using secondary data?
☑ No

Ethics

Population
Check the box indicating the relevant study population.
☐ Students
☑ General population without complaints
☑ General population with specific 'complaints', e.g. stress, medically unexplained complaints
☐ Patients
☐ Other

Age
Check the box indicating the age category of the participants
☑ Younger than 12 years of age
☐ Older than 11 and younger than 16 years of age
☑ 16 years or older

Recruitment
Describe the method of recruitment or selection of participants

Children eligible for this study must be between 6 and 8 years old (inclusive) at the start of the study (August 2025) and must have frequent, daily contact with the participating parent, such as living together. Only one child and one parent per nuclear family may participate, meaning siblings are not included. Children will be excluded if they have a diagnosed psychiatric or neurological disorder as reported by the parent, an intellectual disability with

an IQ below 70, insufficient proficiency in the Dutch language, or if they do not live primarily with the participating parent.

Participants will primarily be recruited through two collaborating practice institutions that support children and parents facing behavioral challenges. These institutions will inform eligible parents via email and one-on-one consultations to ensure clear communication about the study. Additionally, primary schools will reach out to students, parents, and teachers through email, digital communication channels, and posters displayed at schools. Recruitment will focus on schools where children have previously participated in the Dinosaur Child Program, as these schools are already familiar with the intervention. To further expand recruitment, families who have already signed up will be encouraged to invite friends to participate.

This section provides a detailed overview of the procedure to ensure standardized and consistent practices throughout the study:

1. *First contact*: In several ways children and parents get first information about the study (see Recruitment method). Participants first receive a flyer summarizing the information from the letter, along with an information letter containing a QR code to sign up for the study or contact us for further details.

2. *Phone call*: The Dino team conducts a detailed briefing about participation via a phone call before the start of the study. During this call, the following topics are addressed:
    a) Answering all questions from the parents and child
    b) Explaining the procedure in detail
    c) Clarifying the randomization process and the assignment to either the intervention or control condition
    d) Emphasizing the importance of completing all study-related questionnaires, including daily questionnaires, as well as the pre-test and post-test online questionnaires via Qualtrics, which will be sent to parents and teachers
    e) Informing parents that a home visit will take place before the intervention for a child interview and that another home visit will be conducted after the intervention
    f) Addressing any potential concerns
        i. Study duration and intensity (daily questionnaires take 3-5 minutes, and right to withdraw from research)
        ii. Notification times daily questionnaires
        iii. Data privacy (ethical committee approval, compliance with European data processing standards, and pseudonymization of data)
    g) Explaining the reward for participating
    h) Answer remaining questions
    i) Asking parents if they would like to recommend the study to a friend or relative
    j) Informing participants that they can contact the research staff via call, text message or email throughout the study
    k) Providing details about the study start date and next steps
    l) Send informed consent via mail/Whatsapp

3. *Informed consent*: Parents provide active informed consent via an online Qualtrics form administered through Tilburg University. On that form they indicate their intention to participate as well as give additional contact information (e.g. IBAN number) and indicate if they may be contacted for future research.
   Also, children will be informed about the study in an age-appropriate manner and given the opportunity to express their willingness to participate, in addition to the informed consent provided by their parent(s).

4. *Homevisit and online pretest questionnaire:* One month before the start of the intervention, a home visit takes place to conduct a child interview. Additionally, parents and teachers are asked to complete an online questionnaire via Qualtrics to provide baseline information relevant to the study.

5. *Install instruction letter*: Once consent is obtained, participants receive a message thanking them for their participation. They also receive an instruction letter explaining how to install the m-Path app on their smartphone. This letter includes a personal and pseudonymized invitation code, which they must enter in the app to enroll in the study protocol.

6. *Pre-study contact*: One day before the ESM part starts, participants are reminded that the study is about to begin and that they can contact the research staff if they have any remaining questions or will encounter technical difficulties.

7. *Technical support at study start*: On the second day of the ESM data collection participants receive a follow-up message asking if they have encountered any technical difficulties while completing the first daily questionnaire. If needed, the research staff will provide support to resolve any issues.

8. *Ongoing support during study:* Throughout the study, participants have access to a tutorial within the m-Path app, which includes an explanation of the daily diary items. They are encouraged to contact the research team if they have any questions. The Dino team remains available throughout the study period via text message, phone call, or email.

9. *Intermediate evaluation*: After the first few weeks of answering the daily questionnaires participants get a message via whatsapp that we are thankful for their contribution and inquire if they experience any inconvenience or have any questions. Participants are reminded that they can contact the Dino Team any time via text message, call, or mail.

Organization

State the organization where the recruitment of participant will take place

☑ Tilburg University

☑ Other, namely Psychologen Praktijk Timmers B.V. and Stichting Kumbaya

☐ Not applicable, because

Reward
Is there a compensation for participation? If so, state what kind of compensation is offered?
☐ None
☑ Reimbursement of (travel) expenses
☐ Course credit
☑ Financial reward, namely €50,-
☑ Other, namely

Burden
Describe in detail the expected impact and/or potential negative consequences for the participants regarding time and mental and physical impact

Participation in the study is designed to be manageable for families while providing meaningful benefits for children. Time commitment is kept as minimal as possible while ensuring high-quality data collection. The pre-test assessment will take approximately one hour for parents, while the post-test will take around 30 minutes. Additionally, parents will be asked to complete short daily questionnaires, which will take only 3 to 5 minutes per day. For teachers, the pre-test will require approximately 40 minutes, and the post-test around 30 minutes. These assessments are structured to fit within regular routines and are designed to be straightforward and easy to complete.

The intervention itself is designed to be engaging and developmentally appropriate, making participation a positive experience for children. No physical risks are anticipated, and efforts will be made to minimize any potential fatigue by ensuring that sessions remain interactive and enjoyable. To ensure that all children benefit from the program, those in the control condition will be placed on a waitlist and will have the opportunity to participate at a later stage. This guarantees that no child is denied access to an effective intervention. The structured nature of the program provides guidance and support, ensuring that both children and parents feel well-assisted throughout their participation, ultimately fostering positive developmental outcomes.

Protection
Describe the measures that taken to protect the participants (e.g. insurance, debriefing, etc.).

Data will be collected in a **pseudonymized** manner by assigning each participant a unique ID number, ensuring that personal information is stored separately and securely. At the end of the study, participants will receive a debriefing, during which they will be thanked for their participation and compensated for their involvement. Additionally, participants will have the option to contact the research team or, for data collection conducted outside Tilburg University, a relevant institution, counselor, or experienced researcher if they experience any emotional distress related to their participation and require support or further discussion.

Manipulation

Are participants subjected to procedures or experiment-related manipulations or tasks?
☑ Yes

Participants in this study will be subjected to an intervention-related manipulation, as they will be randomly assigned to either the intervention condition or the control condition. Children in the intervention condition will participate in the IY Dinosaur Child Program, a structured and evidence-based intervention designed to improve social and emotional skills in children with (sub)clinical problem behaviors. In contrast, children in the control condition will not receive the intervention immediately but will instead be placed on a waitlist.

To ensure that no child or parent is denied access to an effective intervention, families in the control condition will have the opportunity to participate in the program at a later stage. This waitlist approach ensures that all participants ultimately receive access to the intervention, minimizing ethical concerns related to withholding a proven program. These measures safeguard participants' well-being while maintaining scientific rigor in the study design.

Mandatory documents

Information letter
Is an information letter possible?
☑ Yes

Do you use the standard TSB ERB information letter template?
☑ Yes

Information letter
Is an informed consent possible?
☑ Yes

Do you use the standard TSB ERB informed consent template?
☑ Yes

Additional documents
The following documents must be provided (if applicable):
☑ All surveys/questionnaires that will be used
☐ Description of the stimulus materials
☐ Advertisement
☐ Participants information letter (precedes participation)
☐ Written debriefing
☐ Written consent of organization(s (except Tilburg University) to recruit participants

Data storage

Data storage during research
The following questions relate to the data collection and data storage period. These are dates when all data are collected and analyzed to conduct the research.

Where will the data be stored during the data collection and data analyzing period?
☐ O-drive
☑ Research drive
☐ Surfdrive
☐ SharePoint
☐ Other

Who has access to the raw data during the data collection and data analyzing period?
Rabia Chhangur Principal investigator

Who has access to the processed data during the data collection and data analyzing period?
Eleonore Smalle Co-researcher

Data Archiving period
Data storage during data archiving. The following questions apply to data archiving. After the research is completed and the data is stored in a secure (digital) facility.

Where will the data be archived?
☑ DataVerse
☐ O-drive
☐ Other

Will you archive your data in a data repository other than DataVerse?
☑ No

Who will have access to the raw data during the data archiving period?
Rabia Chhangur Principal investigator

Who will have access to the processed data during the data archiving period?
Eleonore Smalle Co-researcher

Long-term archiving
How long will the data be archived? (In years)
10

Which criteria will you use to decide which data will have to be archived for long-term retention and access? Which (part of the) data will have to be destroyed to ensure privacy protection?

Only data necessary for underpinning scientific publications, as well as data required for verification purposes, including intermediate results, materials, methods of analysis, and workflow details, will be archived and stored.

Should data collected during the study be stored for the required term, of 10-year period, for long-term data retention?

☑ No

Documentation and metadata
What documentation and metadata will be provided and what metadata standard will be used (if any)?

The Research Data Management (RDM) guidelines will be used for organizing data, such as (1) clear folder structure, (2) clear and consistent file names (3) clear version management (original data will be always be preserved in that it is possible to go back to earlier versions), and (4) clear organization, names and labels of variables. Also, the project will be included in the Open Science Framework (OSF). See codebook.

Are you allowed to dispose of the data after the research project ends?
☑ Yes

Non-digital data
Will non-digital data be stored during the study (paper surveys, transcripts, photocopies of original documents)?
☑ No

Data sharing

Data sharing
Will all or part of the data be made available for reuse after completion of the project according to the FAIR Principles?
☑ Yes

When and how will the data be made available for re-use?

All data collected during this project will be archived locally for at least 10 years and made available for re-use via TiU Dataverse. Contact information will only be archived for participants who have explicitly agreed to be contacted for follow-up research. To protect the privacy and confidentiality of participants, including children and their parents, access to the dataset will be restricted and granted only upon request. Requests must include a research proposal that falls outside the scope of the original research line, acknowledgment of the data source, and the signing of a data-sharing agreement outlining the conditions of use and privacy safeguards.

To promote transparency and reproducibility, key research materials, including preregistrations, syntaxes, and codebooks, will be shared via the Open Science Framework (OSF). Each publication will be preregistered unless there are compelling reasons not to do so, and data will only be provided after preregistration of the intended study. All publications will be made fully open access, unless exceptional circumstances prevent this.

Additionally, covariance matrices and syntaxes will be made publicly available after publication to support replication and meta-analyses.

Strict privacy and data protection measures will be in place to ensure the security of participant data. The data will not be reproduced or transferred in any form, meaning that student assistants or other external parties will not have direct access to the dataset. Furthermore, the raw data cannot be accessed without explicit consent from Rabia Chhangur. By implementing these principles, this project balances open science with ethical and legal responsibilities to safeguard participant privacy while fostering transparency in research.

Restrictions
Are there any restrictions for data sharing or any conditions for re-use of the data?
☑ No

Ownership
How will ownership of the data and intellectual property rights to the data be managed? (Explain who will be the owner of the data, meaning who will have the rights to control access.)

Tilburg University will be the owner of the data collected in this study, while the researcher retains the right to manage and control access to the data.

1. External researchers have a meeting with the team to discuss their ideas.
2. The reseachers will check whether there is no conceptual overlap between the work described in this research proposal and external researchers (as already preregistered).
3. The external researcher provides a project description, if possible beforehand.
4. The team discusses with the external researcher the feasibility of the plan (e.g., can questions be answered with the data) and potential overlap with ongoing work of researchers.
5. Iterative improvements are made until questions and hypotheses are agreed upon by each contributing researcher, and the project is considered feasible and unique compared to ongoing work.

Will there be an embargo period for (all or some of the data)?
☑ No

GDPR

Personal data collection and processing
Personal data
By completing out this part of the form you are complying with the GDPR, which requires personal data to be included in Tilburg University's data processing register. This includes a pre-DPIA (Data Protection Impact Assessment), which will reveal whether there are certain risks and whether you are obliged to perform a DPIA.

Which personal data are to be collected and processed?
☐ No personal data will be processed
☑ Yes, namely (multiple answers possible)

General
☑ Contact data (for example name, e-mail address, phone)
☑ Gender
☑ Age
☑ Birthday
☑ Nationality, birth places, birth country
☑ Experience (work education)
☑ Visual materials (pictures, video)

Special data
☐ Biometric data

Legal base
What is the lawful basis on which the processing activity takes place?
☑ (1) Consent
☐ (2) Legitimate interest as scientific researcher (*gerechtvaardigd belang)
☐ (3) Permission

Anonymization
After collection, will data be anonymized or pseudonymized? And if so, who will have access to the identifying file?

Data will be pseudonymized. The keyfile will be stored separately form the data and will be only accessible thy the principal investigator. Contact information will only be stored for participants who agreed to be contacted for follow-up research. Only Rabia Chhangur (Principal Investigator) will have acces to the identifying file.

To protect this data, all data will be pseudonymization with secure storage methods. Data will be processed in compliance with GDPR, which may include conducting a Data Protection Impact Assessment (DPIA) and obtaining explicit consent from participants. GDPR compliance is assessed during the combined ethical, GDPR and data management process of the university.

Processors
Are there any external parties (processors) involved in this study regarding data collection, data storage, archiving and/or other data-related activities? If so, please describe and name them here and state the website(s) of the processor(s) and/or texts.

Data collection
☐ Not applicable
☑ Yes, namely

Psychologen Praktijk Timmers B.V. and Stichting Kumbaya will conduct child interviews under a contract with Tilburg University (TiU). A formal agreement is established to outline the terms of collaboration. TiU remains responsible for data storage, processing, and archiving, ensuring compliance with the General Data Protection Regulation (GDPR**)** and the Dutch Code of Conduct for Research Integrity (UNL, 2018)**.**

External Processors:

- Psychologen Praktijk Timmers B.V. – Data collection
- Stichting Kumbaya – Data collection

All data will be securely managed according to TiU's data management policies.

Data storage
☑ Not applicable
☐ Yes, namely

Data archiving
☑ Not applicable
☐ Yes, namely

Other data-related activities e.g., analyses
☑ Not applicable
☐ Yes, namely

Third parties
Will you receive personal data from or provide personal data to a third party and which organization will determine the purpose for and means of the processing?
☑ yes

Which third parties (data controllers and processors) are the data provided to by default?

Psychologen Praktijk Timmers B.V. and Stichting Kumbaya will conduct child interviews under a contract with Tilburg University (TiU). The collected data will be pseudonymized, with the principal investigator retaining a separate key that allows re-identification. Consequently, the data remains classified as personal data under the GDPR.

TiU will receive the pseudonymized personal data from Psychologen Praktijk Timmers B.V. and Stichting Kumbaya. As TiU determines both the purpose and the essential means of processing—including data collection, storage, security measures, and research use—it assumes the role of data controller. TiU ensures compliance with data protection regulations, while Psychologen Praktijk Timmers B.V. and Stichting Kumbaya act as data processors, handling the data strictly according to TiU's instructions.

Dino studie

DINO studie

Dagelijks en **I**nterventiegericht onderzoek voor het **N**avigeren en **O**ntwikkelen van kansen

Deze brief bevat belangrijke informatie over dit onderzoek waaraan u en uw kind kunnen deelnemen. Lees deze brief zorgvuldig door. U mag altijd vragen stellen voordat u beslist of u wilt deelnemen.

Wat houdt het onderzoek in?
Deze studie onderzoekt waarom sommige kinderen profiteren van interventies terwijl andere kinderen hier meer moeite mee hebben. Wij gebruiken het bewezen effectieve *Incredible Years* Dinoschool programma om te onderzoeken waarom het programma bij sommige kinderen nog veel beter werkt dan bij andere kinderen.

De Dinoschool is een speelse interventie die kinderen van 4 t/m 8 jaar met dwars en opstandig gedrag helpt hun sociale, emotionele en cognitieve vaardigheden te verbeteren. De interventie wordt in groepsverband uitgevoerd met 5 tot 6 kinderen, verspreid over 18-wekelijkse lessen van 2 uur.

Uw kind kan worden ingedeeld in een van de volgende twee groepen:

1. **Interventiegroep:** Deze groep volgt de Dinoschool interventie.
2. **Controlegroep:** Deze groep ontvangt geen interventie en volgt de standaardzorg zoals deze normaal wordt aangeboden. Uw kind kan op een wachtlijst gezet worden om na afloop van het onderzoek alsnog mee te doen met de Dinoschool.

Alle gezinnen worden willekeurig verdeeld in twee groepen om verschillen te voorkomen. De toewijzing gebeurt door loting, die de onderzoekers niet kunnen beïnvloeden. De helft van de gezinnen wordt ingedeeld in groep 1. Deze kinderen worden uitgenodigd om deel te nemen aan de Dinoschool. Met deze interventie willen we de sociale, emotionele en cognitieve vaardigheden van kinderen versterken en dwars en opstandig gedrag verminderen.

Om achteraf te kunnen bepalen of eventuele veranderingen daadwerkelijk door de interventie zijn veroorzaakt, is het nodig ook een controlegroep in het onderzoek te betrekken. Deze kinderen vormen groep 2. Zonder een controlegroep kunnen we namelijk niet met zekerheid vaststellen of de Dinoschool heeft bijgedragen aan de veranderingen in positief gedrag. Groep 2 neemt daarom niet deel aan de interventie. Aan beide groepen worden wel dezelfde vragen gesteld en beide groepen ontvangen dezelfde vergoeding. Er is altijd de mogeljkhed om na afloop van het onderzoek alsnog de interventie te volgen.

Wat houdt deelname aan het onderzoek in?
Het onderzoek loopt over een periode van ongeveer zes maanden en bestaat uit twee meetrondes en een periode waarin u dagelijks een korte vragenlijst invult. In totaal zullen ongeveer 120 gezinnen deelnemen.

Tijdens deze periode vragen wij het volgende van u:

- Twee keer een online vragenlijst invullen, in augustus 2025 (voor de interventie) en februari 2026 (na afloop). Elke vragenlijst duurt ongeveer 60 minuten en gaat over uw ervaringen en het gedrag van uw kind.

- Twee huisbezoeken van ongeveer 30 minuten, waarbij een medewerker op speelse wijze een korte taak uitvoert met uw kind om inzicht te krijgen in zijn of haar emoties en sociale vaardigheden.
- Dagelijks een korte vragenlijst invullen via een app, samen met uw kind. Deze vragenlijst duurt 3 tot 5 minuten per dag en wordt ingevuld gedurende 20 weken: 1 week voorafgaand aan de interventie, 18 weken tijdens de interventie en 1 week erna.

De totale tijdsinvestering bedraagt ongeveer 10 tot 15 uur, verspreid over de gehele onderzoeksperiode. Voor uw tijd en inzet kunt u tot ongeveer €50,- verdienen, afhankelijk van uw deelname aan de verschillende onderdelen van het onderzoek (zie ook het kopje 'Is er een Vergoeding').

Wat houdt deelname aan de Dinoschool in?
De Dinoschool is een speels en educatief programma voor kinderen van 6 t/m 8 jaar. Het programma helpt kinderen hun sociale, emotionele en cognitieve vaardigheden te ontwikkelen. De focus ligt op het vergroten van zelfvertrouwen, samenwerken, omgaan met emoties en het oplossen van problemen. Kinderen in de interventiegroep volgen 18 wekelijkse groepslessen. Deze lessen zijn leuk en interactief, met veel aandacht voor leren door middel van spel en verhalen met puppets. Elke les duurt ongeveer twee uur en vindt plaats in een veilige omgeving bij u in de buurt, onder gebeleiding van getrainde professionals.

Wat zijn de voordelen en nadelen van deelname aan het onderzoek?
Deelname aan het onderzoek is geheel vrijwillig en brengt geen nadelen met zich mee, behalve dat het tijd kost. De eerste meetronde bestaat uit een aantal online vragenlijsten van ongeveer één uur en een huisbezoek van ongeveer 30 minuten. Daarnaast vult u samen met uw kind dagelijks een korte vragenlijst in via een app. Deze korte vragenlijst duurt ongeveer 3-5 minuten per keer. Voor uw tijd en inspanning kunt u een vergoeding tot maximaal €50, verdeeld over drie onderdelen: €10 voor het invullen van de vragenlijsten aan het begin van het onderzoek, €15 aan het einde, en tot ongeveer €25 voor de dagelijkse korte vragenlijsten (€0,15 per ingevulde dag).

Mogelijk wordt uw kind uitgenodigd om deel te nemen aan de Dinoschool (wanneer hij of zij wordt ingedeeld in groep 1). Deelname aan de Dinoschool is volledig kosteloos. Uit eerder onderzoek blijkt dat kinderen na het volgen van de Dinoschool hun sociale, emotionele en cognitieve vaardigheden verbeteren en minder dwars en opstandig gedrag vertonen.

Als uw kind niet wordt ingedeeld in groep 1 en de interventie niet volgt, staat het u uiteraard vrij om tijdens het onderzoek andere vormen van hulp even andere kindinterventie te zoeken. Wij helpen u daar desgewenst graag bij. Ook kan uw kind indien gewenst, op de wachtlijst worden geplaatst om na afloop van het onderzoek alsnog de Dinoschool te volgen.

Is deelname aan het onderzoek vrijwillig?
Wij vragen uw toestemming voor deelname aan het onderzoek gedurende de looptijd van dit onderzoek. Deelname aan dit onderzoek is vrijwillig. U hebt het recht om niet deel te nemen aan deze studie. Als u tijdens het onderzoek besluit te willen stoppen met uw deelname, kan dit op elk moment zonder enige negatieve consequenties en zonder opgaaf van reden. U bent vrij om vragen of taken over te slaan waar u zich niet comfortabel bij voelt

De onderzoekers kunnen het onderzoek te allen tijde beëindigen. De beslissing om het experiment te beëindigen kan worden gemaakt om uw gezondheid en veiligheid te beschermen, of omdat het onderzoeksplan voorschrijft dat mensen die niet voldoen aan bepaalde voorwaarden of de instructies niet volgen, niet kunnen deelnemen.

Wat zijn mijn rechten? [Voor persoonlijke data]
Als participant heeft u het recht op inzage, rectificatie, vergetelheid, beperking of voorkomen van verwerking van uw persoonlijke gegevens. Voor meer informatie, zie: https://www.tilburguniversity.edu/nl/privacy

Wat gebeurt er met mijn gegevens?
Alle verzamelde informatie over u en uw kind zal vertrouwelijk worden behandeld. Uw onderzoeksgegevens worden gepseudonimiseerd (dat wil zeggen dat alle informatie die direct aan u gekoppeld kan worden, wordt vervangen door een codenaam of -nummer die alleen bekend is bij het onderzoeksteam). Informatie die u persoonlijk identificeert, wordt niet vrijgegeven zonder uw schriftelijke toestemming. De onderzoeksresultaten die worden gepubliceerd zullen op geen enkele wijze vertrouwelijke informatie of persoonlijke gegevens van of over uw bevatten waardoor iemand u kan herkennen, tenzij u via ons toestemmingsformulier expliciet toestemming heeft gegeven voor het vermelden van uw naam.

Is er een vergoeding?
Voor uw tijd en inzet kunt u een vergoeding tot maximaal €50,- ontvangen, verdeeld over drie onderdelen: het invullen van de vragenlijsten aan het begin (€10) en einde (€15) van het onderzoek, en de dagelijkse korte vragenlijsten gedurende de interventieperiode (€0,15 per ingevulde dag, tot ongeveer €25). De vergoeding is afhankelijk van de hoeveelheid ingevulde vragenlijsten en wordt aan het einde van het onderzoek uitbetaald. Deelname aan alle onderdelen is dus niet verplicht om (gedeeltelijke) vergoeding te ontvangen.

Wat is de opslagtermijn van de onderzoeksgegevens?
De gepseudonimiseerde onderzoeksgegevens zullen veilig worden opgeslagen voor een periode van 10 jaar. De geanonimiseerde gegevens (dat wil zeggen dat alle informatie die aan u persoonlijk kan worden gekoppeld, wordt verwijderd) die in dit onderzoek zijn verzameld, kunnen nuttig zijn voor vervolgonderzoek of andere onderzoeken en zullen hiervoor anoniem beschikbaar gemaakt worden, bijvoorbeeld door deze gegevens te delen met collega-onderzoekers of beschikbaar te stellen via een (openbaar) data-archief of repository. Vanaf dat moment is het niet meer mogelijk om uw gegevens te verwijderen. Als u uw gegevens wilt verwijderen, kunt u tot en met juni 2026 contact opnemen met het onderzoeksteam.

Hoe worden onderzoeksgegevens verwerkt?
De onderzoeksgegevens worden veilig opgeslagen. De gegevens worden gepseudonimiseerd, waarbij de sleutel apart en alleen toegankelijk is voor de hoofdonderzoeker. Contactgegevens worden alleen bewaard als u toestemming heeft gegeven voor vervolgonderzoek. De verwerking van gegevens voldoet aan de AVG en wordt regelmatig gecontroleerd door de universiteit.

Is er goedkeuring verkregen van de Ethische Toetsingscommissie?
Deze studie is goedgekeurd door de ethics review board (ERB) van Tilburg University onder dossiernummer TSB_RP2172.

Hoe kan ik deelnemen?
Als u wilt deelnemen aan dit onderzoek, kunt u daarvoor toestemming geven door de onderstaande QR-code te scannen, op de bijgevoegde link te klikken of een e-mail te sturen naar dr. Rabia Chhangur: https://tilburgss.co1.qualtrics.com/jfe/form/SV_9XiRlwUKr6cfgc6

Hoe kan ik met jullie contact opnemen?
Voor vragen over dit onderzoek kunt u contact op nemen met:
Dr. Rabia Chhangur: R.R.Chhangur@tilburguniversity.edu

Als u vragen, zorgen of bezwaren heeft die u aan iemand anders dan de onderzoeker wilt melden, neem dan contact op met de Ethics Review Board van Tilburg School of Social and Behavioral Sciences via e-mail erb@tilburguniversity.edu.

DINO studie

Dagelijks en **I**nterventiegericht onderzoek voor het **N**avigeren en **O**ntwikkelen van kansen

Beste leraar,

U ontvangt deze brief omdat de ouders van een leerling uit uw klas hebben aangegeven deel te willen nemen aan de DINO studie (Dagelijks en Interventiegericht onderzoek voor het Navigeren en Ontwikkelen van kansen).

Wat houdt het onderzoek in?
In deze studie onderzoeken we waarom sommige kinderen meer baat hebben bij gedragsinterventies dan andere kinderen. We doen dit aan de hand van het bewezen effectieve *Incredible Years* Dinoschool-programma, een speelse training voor kinderen van 6 t/m 8 jaar met opstandig gedrag. De training bestaat uit 18 wekelijkse groepslessen van 2 uur, gericht op het versterken van sociale, emotionele en cognitieve vaardigheden.

Kinderen worden willekeurig ingedeeld in een van twee groepen:

1. De interventiegroep volgt de Dinoschool.
2. De controlegroep ontvangt (nog) geen interventie, maar behoudt de mogelijkheid om na het onderzoek alsnog deel te nemen.

Wat vragen we van u als leraar?
Omdat u als leerkracht een belangrijke rol speelt in de ontwikkeling van het kind, vragen wij u om op twee momenten in het schooljaar enkele korte vragenlijsten in te vullen over het gedrag en functioneren van de leerling:

1. **In september 2025** (voor de start van de interventie)
2. **In februari 2026** (na afloop van de interventieperiode)

De vragenlijsten bestaan uit de volgende onderdelen:

- Vragenlijst voor Leerkrachten (TRF)
- Gedragsvragenlijst voor Executieve Functies – Leerkrachtversie (BRIEF)
- Schaal voor Sociale Vaardigheden – Leerkrachtversie

Het invullen van deze vragenlijsten kost in totaal ongeveer 30 tot 45 minuten per meetmoment. U ontvangt de vragenlijsten digitaal via een beveiligde **link** die we per e-mail toesturen. We vragen u vriendelijk om de vragenlijsten **binnen één week na ontvangst** in te vullen. U ontvangt hiervoor nog instructies en indien gewenst begeleiding vanuit het onderzoeksteam.

Waarom uw bijdrage belangrijk is?
Uw observaties als leraar geven ons waardevolle informatie over hoe het kind functioneert in de schoolcontext. Daarmee draagt u bij aan wetenschappelijk inzicht in hoe en bij wie gedragsinterventies zoals Dinoschool het meest effectief zijn.

Is deelname aan het onderzoek vrijwillig?
Deelname aan dit onderzoek is vrijwillig. U hebt het recht om niet deel te nemen aan deze studie. Als u tijdens het onderzoek besluit te willen stoppen met uw deelname, kan dit op elk moment

zonder enige negatieve consequenties en zonder opgaaf van reden. U bent vrij om vragen of taken over te slaan waar u zich niet comfortabel bij voelt

De onderzoekers kunnen het onderzoek te allen tijde beëindigen. De beslissing om het experiment te beëindigen kan worden gemaakt om uw gezondheid en veiligheid te beschermen, of omdat het onderzoeksplan voorschrijft dat mensen die niet voldoen aan bepaalde voorwaarden of de instructies niet volgen, niet kunnen deelnemen.

Wat gebeurt er met mijn gegevens?
Alle verzamelde informatie over uw leerling worden vertrouwelijk worden behandeld. Uw onderzoeksgegevens worden gepseudonimiseerd (dat wil zeggen dat alle informatie die direct aan u gekoppeld kan worden, wordt vervangen door een codenaam of -nummer die alleen bekend is bij het onderzoeksteam). Informatie die u persoonlijk identificeert, wordt niet vrijgegeven. De onderzoeksresultaten die worden gepubliceerd zullen op geen enkele wijze vertrouwelijke informatie of persoonlijke gegevens van of over uw bevatten waardoor iemand u kan herkennen.

Wat is de opslagtermijn van de onderzoeksgegevens?
De gepseudonimiseerde onderzoeksgegevens zullen veilig worden opgeslagen voor een periode van 10 jaar. De geanonimiseerde gegevens (dat wil zeggen dat alle informatie die aan u persoonlijk kan worden gekoppeld, wordt verwijderd) die in dit onderzoek zijn verzameld, kunnen nuttig zijn voor vervolgonderzoek of andere onderzoeken en zullen hiervoor anoniem beschikbaar gemaakt worden, bijvoorbeeld door deze gegevens te delen met collega-onderzoekers of beschikbaar te stellen via een (openbaar) data-archief of repository. Vanaf dat moment is het niet meer mogelijk om uw gegevens te verwijderen. Als u uw gegevens wilt verwijderen, kunt u tot en met juni 2026 contact opnemen met het onderzoeksteam.

Hoe worden onderzoeksgegevens verwerkt?
De onderzoeksgegevens worden veilig opgeslagen. De gegevens worden gepseudonimiseerd, waarbij de sleutel apart en alleen toegankelijk is voor de hoofdonderzoeker. Contactgegevens worden alleen bewaard als u toestemming heeft gegeven voor vervolgonderzoek. De verwerking van gegevens voldoet aan de AVG en wordt regelmatig gecontroleerd door de universiteit.

Is er goedkeuring verkregen van de Ethische Toetsingscommissie?
Deze studie is goedgekeurd door de ethics review board (ERB) van Tilburg University onder dossiernummer TSB_RP2172.

Vragen of meer informatie?
Mocht u vragen hebben over het onderzoek of uw rol hierin, dan kunt u contact opnemen met de hoofdonderzoeker, dr. Rabia Chhangur via R.R.Chhangur@tilburguniversity.edu. Wij danken u hartelijk voor uw medewerking!

Met vriendelijke groet,

Het DINO-onderzoeksteam
Tilburg University

DINO studie:

Dagelijks en **I**nterventiegericht onderzoek voor het **N**avigeren en **O**ntwikkelen van kansen bij jonge kinderen

Onderzoekers:
Dr. Rabia Chhangur, Departement Ontwikkelingspsychologie, Tilburg University
Dr. Eleonore Smalle, Departement Ontwikkelingspsychologie, Tilburg University

Handtekening

Met uw handtekening geeft u vrijwillig en bewust toestemming voor deelname aan dit onderzoek. U ziet hiermee niet af van uw wettelijke rechten om uw deelname te allen tijde in te mogen trekken.

Met uw handtekening geeft u tevens aan dat u de informatiebrief behorende bij dit onderzoek in zijn geheel heeft gelezen, en dat u akkoord gaat met de volgende punten:

- Ik heb de informatiebrief behorende bij dit onderzoek in zijn geheel gelezen en begrepen.
- Ik kon eventueel aanvullende vragen stellen over het onderzoek en deze vragen zijn voldoende beantwoord.
- Ik heb voldoende tijd gehad om te beslissen of ik wil deelnemen aan dit onderzoek.
- Ik weet dat deelname aan dit onderzoek geheel vrijwillig is.
- Ik weet dat het onderzoek loopt van augustus 2025 t/m maart 2026.
- Ik weet dat ik op ieder moment kan beslissen om af te zien van deelname, zonder negatieve gevolgen, en dat ik hiervoor geen reden hoef op te geven.
- Ik weet dat ik het recht heb op inzage, rectificatie, vergetelheid, beperking of voorkomen van verwerking van mijn persoonlijke gegevens.
- Ik weet dat mijn gegevens zullen worden verwerkt zoals aangegeven in de informatiebrief en dat alleen het onderzoeksteam mijn gegevens kunnen inzien.
- Ik geef toestemming om mijn onderzoeksgegevens te gebruiken voor de doelen die in de informatiebrief staan.
- Ik geef toestemming om mijn geanonimiseerde onderzoeksgegevens op te slaan voor de wettelijke periode van 10 jaar.

Ik verleen hierbij vrijwillig en bewust toestemming voor deelname aan het onderzoek:

Dino studie

Voor- en achternaam kind: ____

Geboortedatum kind: ____

Deelnemende ouder

Datum: ____________________ Handtekening: ______________________

Mijn persoonlijke gegevens:

Voor- en achternaam: ____

Adres: ____

Postcode: _____________________Woonplaats: _______________________________________

Telefoonnummers: ____

E-mailadres: ____

Bankrekeningnummer (IBAN): ____

Partner (indien van toepassing):

Voor- en achternaam: ____

Adres: ____

Postcode: _____________________Woonplaats: _______________________________________

Telefoonnummers: ____

E-mailadres: ____

Uw voorkeuren

In de toekomst vinden mogelijk nog vervolgonderzoeken plaats.
Mogen we u daarvoor te zijner tijd opnieuw benaderen? ☐ **Ja** ☐ **Nee**
Mogen wij foto's en video's die gemaakt worden tijdens het onderzoek gebruiken voor...

- Onderwijs, bijvoorbeeld voor psychologie studenten ☐ **Ja** ☐ **Nee**
- Onderzoek presentaties, zoals op congressen ☐ **Ja** ☐ **Nee**
- Sociale media, zoals onze Facebook pagina ☐ **Ja** ☐ **Nee**
